# Where is Patient Safety Research and Practice Heading? A Response to Recent Commentaries

**DOI:** 10.15171/ijhpm.2018.112

**Published:** 2018-11-21

**Authors:** Russell Mannion, Jeffrey Braithwaite

**Affiliations:** ^1^Health Services Management Centre, University of Birmingham, Birmingham, UK.; ^2^Australian Institute of Health Innovation, Macquarie University, Sydney, NSW, Australia

We appreciate greatly the scholarship and inventive debate stimulated by the authors offering a commentary to our article: *False Dawns and New Horizons in Patient Safety Research and Practice.*^1^ Each commentary engaged critically and imaginatively with the main ideas we presented, offering some degree of affirmation of our argument, but also taking the discussion in new directions, theoretically and empirically. Taken together, the commentaries have helped to broaden our thinking in relation to patient safety research and practice, and, we hope, that of the readers of the *Journal*. Here, we offer a rejoinder to those commentaries and then summarise how, as we read their views, they have provided pointers to what we should do next.

In their contribution, Andrew Carson-Stevens, Liam Donaldson, and Aziz Sheikh^2^ are largely sympathetic to our call for a shift towards incorporating Safety-II thinking into patient safety research and practice (focusing on what goes right as well as what goes wrong). They make a strong case for the ongoing value and continuing salience of some Safety-I based approaches (which we also support) and that health care leaders should proactively and simultaneously seek signals for improvement from unsafe and deficient, but also good, everyday care. They contend that the key difficulty with existing Safety-I approaches is that few reliable ways have been found to strengthen how learning from errors is used in health systems to improve quality of care and argue that the key challenge is that incident reporting systems capture essential information that can inform improvement efforts, but have not yet been used to full advantage.

Rebecca Lawton in her piece echoes the point that Safety-I approaches *per se* should not be blamed for the lack of progress in patient safety outcomes, but rather how these approaches have so far been operationalised in health care organisations and systems.^3^ Therefore, rather than the founding principles of systems thinking being fundamentally flawed she highlights that the key problem to address is the poor-quality of existing incident investigations and a failure to act on the information provided. She warns that unless operationalised in the right way, Safety-II may merely serve to perpetuate the problems of existing Safety-I approaches by focusing on ‘excellent’ practice that others must aspire to, with new rules and protocols imposed in an attempt to replicate more ‘excellence.’

Changing the pace, Kelly Smith and Annette Valenta in their commentary caution that the shift from Safety-I to Safety-II may be insufficient, in and of itself, to improve patient safety outcomes.^4^ They note that many of those currently working in patient safety and quality are formally trained in medicine, nursing, law, pharmacy, and healthcare administration, but lack training in patient safety. In light of this, they argue that the key problem is the absence of a commonly understood set of core competencies (knowledge, skills, and attitudes) among those working in patient safety; and they further maintain that until the field of patient safety unites around these competencies and requires certification as a profession, neither Safety-I nor Safety-II will reach its full potential.

Mark Sujan’s perspective on patient safety is probably closest in spirit to ours.^5^ He argues that current approaches to learning in health organisations only consider the few extraordinary situations of systems failure; they are essentially focussed, or even obsessed, with things going wrong. He contends that while such insights can be useful, they are barely adequate to generate a comprehensive understanding of safety in complex adaptive systems.^6^ Drawing inspiration from resilient engineering and resilient health care.^7-10^ He argues that we need to study the adaptations that health care workers make in their everyday work. In his own research, Sujan shows the importance of exploring the narratives that staff articulate about their daily “hassles” and how they adapt to these problems, all the while continuing to deliver safe care. He sees that it is the ability of healthcare workers and clinical systems to adapt and to make dynamic trade-offs which enable safe care to be delivered in the face of constant disturbances, tensions and contradictions, and competing organisational priorities. In other words, a Safety-II approach to organisational learning enables us to learn about why, most of the time, things go right, through the myriad circumstances of flexing and adapting in the system. It is this that prevents everyday disturbances and disruptions from becoming everyday catastrophes.

Changing focus yet again, Joanne Travaglia in her commentary^11^ applies a sociological lens, developing the notion that if Safety-II is to succeed where Safety-I failed, it needs to question not only the ontology, but the epistemology and ethics of the patient safety movement. Taking the Bourdieusian perspective which she so persuasively applied years ago in her doctoral studies, she challenges the taken for granted – what Bourdieu would call the doxa of the patient safety movement – and takes to task the positioning of the current patient safety movement as a natural progression from the disciplining of the individual to the disciplining of the system. For Travaglia, the continued impact of managerial and professional power relations which manifests in healthcare generally, and patient safety particularly, must be addressed. We share her sentiments, even if we are not so sure how to equalise power relations in a system as deeply entrenched, political and hierarchical as healthcare.

## Towards a New Research and Practice Agenda

Where, then, does this analysis take us? The commentators offer an elaboration of our original article. Some things we agree on. There is a universal pact amongst us that the present approach, whether or not everyone is happy to label it Safety-I, is inadequate. There does seem to be a need to develop better systems to capture and spread learning. For Carson-Stevens et al this takes the form of incident reporting and learning systems, and for Sujan it is organisational learning. Smith and Valenta think the right path can be taken with skills development, particularly enhanced competencies for all. And Lawton, while defending some of the earliest thinkers in patient safety who did indeed have a sophisticated view of systems and complexity (but many others did not), strongly supports a bottom-up approach with involvement of staff and patients in a thoroughgoing way. And the caution of Travaglia is timely in this era of major political upheaval—that we must be mindful that there are deeply encrusted, taken for granted power imbalances that are woven tightly into the fabric of healthcare organisation and delivery.

Putting this together, a revitalised research agenda, designed to strengthen current patient safety approaches, might be centrally concerned with systems, organisational and individual learning and its associated politics and power relations, focused on bottom-up contributions by staff and patients who have renewed competencies in both Safety-I and Safety-II.

We see much to like here, but would simply urge all researchers to factor in a key point about Safety-II: healthcare work is always peppered with distractions and interruptions, and much clinical work is about addressing normal, naturally-occurring everyday troubles. People doing the work (work-as-done) are in the loop of safety and can make corrections, but those prescribing solutions (doing work-as-imagined) are not in the loop and cannot correct in real time. This includes commentators to this series, policymakers and managers, and researchers, too.

In the end, we cannot stress enough that on-the-ground resilient solutions always emerge from workaround mechanisms like flexing, adjusting, modifying and fudging. So we would like to add to any research agenda that we need a more complete understanding of how those working on the front lines of care increase their adaptive capacity, handle and buffer against cognitive overload, manoeuvre everyday difficulties and navigate through complexity, deploying their situational awareness to keep patients mostly safe, most of the time. We should have as a core to our patient safety activities not the top-down admonition to the coalface “this is what you need to do” but rather “where are you trying to go and how can we help you get there.”

## Ethical issues

Not applicable.

## Competing interests

Authors declare that they have no competing interests.

## Authors’ contributions

Both authors contributed equally to the writing of this article.

## Funding

RM is funded by a number of NIHR grants and programmes. He is also a member of the community of scholars of the Healthcare Improvement Studies (THIS) Institute led by the University of Cambridge. JB is funded by multiple NHMRC grants including the Partnership Centre for Health System Sustainability (APP9100002) and an NHMRC Program Grant (APP1054146).

## Authors’ affiliations

^1^Health Services Management Centre, University of Birmingham, Birmingham, UK. ^2^Australian Institute of Health Innovation, Macquarie University, Sydney, NSW, Australia.
